# Foot Drop: An Anatomical, Clinical, and Electrodiagnostic Approach to Localization

**DOI:** 10.26502/fjsrs00101

**Published:** 2026-08-26

**Authors:** Bahram Saber, Devendra K. Agrawal

**Affiliations:** 1 Department of Translational Research, College of Osteopathic Medicine of the Pacific, Western University of Health Sciences, Pomona CA 91766, USA.

**Keywords:** Common Fibular Neuropathy, Common Peroneal Neuropathy, Electrodiagnostic Evaluation, Electromyography (EMG), Foot Drop, L5 Radiculopathy, Lumbosacral Plexopathy, Nerve Conduction Studies (NCS), Neuromuscular Localization, Peripheral Nerve Entrapment, Sciatic Neuropathy

## Abstract

Foot drop, characterized by weakness of ankle and toe dorsiflexion, is a clinical sign rather than a diagnosis and requires accurate neuroanatomic localization. Potential sites of injury include the L5 nerve root, lumbosacral plexus, sciatic nerve, and common fibular (peroneal) nerve. Because lesions at different levels can produce overlapping motor and sensory findings, incorrect localization may lead to inappropriate diagnostic testing or treatment. This narrative review presents an anatomical, clinical, and electrodiagnostic framework for localizing neurogenic foot drop, with particular emphasis on distinguishing L5 radiculopathy, common fibular neuropathy at the fibular head, fibular-division predominant sciatic neuropathy, and lumbosacral plexopathy. High yield examination findings include ankle inversion and hip abduction strength, while electrodiagnostic localization relies on strategically selected sensory and motor nerve conduction studies and needle electromyography of muscles representing competing anatomic levels. The short head of the biceps femoris is particularly useful for distinguishing a proximal fibular division sciatic lesion from a typical common fibular neuropathy at the fibular head. Ultrasound and magnetic resonance imaging provide complementary structural information when focal nerve pathology is suspected. Integrating examination, electrodiagnostic testing, and targeted imaging provides a systematic approach to localization and guides subsequent etiologic evaluation and management.

## 1. Introduction

Foot drop, typically characterized by weakness of ankle dorsiflexion and often accompanied by weakness of toe extension, may produce a steppage gait or foot slap. It is a clinical sign rather than a single diagnosis and is encountered across orthopedic, spine, rehabilitation, and neurologic practice [1,2]. Neurogenic foot drop can arise at several anatomic levels, including the L5 nerve root, lumbosacral plexus, sciatic nerve, and common fibular nerve, with common fibular neuropathy frequently occurring near the fibular head or neck [2,3].

Accurate localization is important because abnormalities at different levels can produce substantially overlapping clinical presentations. Common fibular neuropathy may resemble L5 radiculopathy, while preferential involvement of the fibular division in sciatic neuropathy may mimic a lesion at the fibular head. Concurrent lesions, including L5 radiculopathy and common fibular entrapment, may further complicate localization [3,4]. A localization-first approach can therefore reduce diagnostic anchoring and direct electrodiagnostic testing and imaging toward the appropriate anatomic level.

This narrative review organizes the evaluation of foot drop around the course of the relevant neural pathway, clinical findings that discriminate among competing localizations, electrodiagnostic strategies for testing these hypotheses, and the complementary role of structural imaging.

## 2. Anatomy of the Pathway

The motor pathway relevant to peripheral neurogenic foot drop begins with lumbosacral nerve roots that contribute to the lumbosacral plexus and ultimately to the sciatic nerve. Fibular division fibers within the sciatic nerve arise predominantly from L4 to S1 contributions. The tibial and fibular fascicular divisions remain anatomically distinct within the sciatic nerve well proximal to its terminal bifurcation. The sciatic nerve usually divides near the popliteal fossa, although more proximal division may occur [3,5].

The common fibular nerve courses laterally and becomes superficial as it passes around the fibular neck, making it particularly susceptible to compression and trauma. It subsequently divides into the deep and superficial fibular nerves. The deep fibular nerve supplies the principal ankle and toe dorsiflexors and provides sensation to the first dorsal web space. The superficial fibular nerve supplies the principal ankle evertors and provides cutaneous sensation to the distal anterolateral leg and most of the dorsum of the foot, excluding the first dorsal web space and territories supplied by the saphenous and sural nerves [3,5,6].

The short head of the biceps femoris has specific localizing value because it receives innervation from the fibular division of the sciatic nerve proximal to the knee. In a typical common fibular neuropathy localized to the fibular head or neck, this muscle is expected to be spared. Electrodiagnostic abnormality in the short head of the biceps femoris therefore supports a lesion proximal to its motor branch, including fibular-division-predominant sciatic neuropathy [5,7,8,9].

## 3. Differential Diagnosis by Anatomic Level

### a. L5 Radiculopathy

L5 radiculopathy commonly causes weakness of ankle dorsiflexion and great toe extension. Unlike an isolated common fibular neuropathy, an L5 lesion may also impair ankle inversion through involvement of tibialis posterior, an L5-predominant muscle innervated by the tibial nerve. Weakness of hip abduction through involvement of gluteus medius provides another finding outside the common fibular nerve distribution that may support an L5 level process. Sensory symptoms may involve the lateral leg and dorsum of the foot toward the great toe, although dermatomal sensory abnormalities are variable. Low back pain, radiating leg pain, and nerve tension maneuvers may provide additional evidence of a radicular process [2,3,10].

### b. Common Fibular Neuropathy at the Fibular Head

Common fibular neuropathy classically produces weakness of ankle dorsiflexion, toe extension, and ankle eversion while sparing ankle inversion and plantar flexion. Sensory symptoms may involve the anterolateral distal leg and dorsum of the foot, while the plantar surface is typically spared. The superficial location of the nerve at the fibular head and neck makes this segment vulnerable to external compression and injury. Associated settings include habitual leg crossing, prolonged positioning, substantial weight loss, fibular head or knee trauma, and structural lesions such as ganglion cysts or osteochondromas [3,11,12].

### c. Fibular-Division-Predominant Sciatic Neuropathy

The fibular division may be disproportionately affected in sciatic neuropathy, allowing a proximal sciatic lesion to closely resemble common fibular neuropathy. Sciatic neuropathy may follow hip surgery, external compression, injection injury, or pelvic or proximal thigh trauma [7,13,14]. When clinical and distal electrodiagnostic findings appear predominantly fibular, proximal needle examination becomes particularly important. Abnormality in the short head of the biceps femoris supports localization proximal to the common fibular nerve at the fibular head and should prompt consideration of a sciatic lesion [5,7,8,13].

### d. Lumbosacral Plexopathy

Lumbosacral plexopathy generally produces a multiroot, multinerve pattern of weakness and sensory abnormality that cannot be explained by a single nerve root or peripheral nerve. The pattern varies with the portion of the plexus affected. Pelvic or retroperitoneal malignancy, hematoma, radiation, surgery, and trauma are important etiologic considerations [2,10]. Electrodiagnostic demonstration of abnormalities spanning multiple peripheral nerves and root levels, particularly with sensory nerve action potential abnormalities and without corresponding paraspinal denervation, can support a plexus level localization.

### e. Central and Other Nonperipheral Causes

Not all foot drop is caused by a peripheral nerve, plexus, or nerve root lesion. Parasagittal cortical lesions, subcortical stroke, and spinal cord disease may produce dorsiflexion predominant weakness. Upper motor neuron findings such as hyperreflexia, spasticity, an extensor plantar response, or more widespread neurologic abnormalities should prompt evaluation beyond the peripheral localization framework [2,10].

More diffuse disorders should also be considered when weakness is bilateral, slowly progressive, or accompanied by broader neurologic abnormalities. These include generalized and hereditary peripheral neuropathies, motor neuron disease, and selected neuromuscular disorders. Clinical context therefore remains important even when the presenting deficit appears anatomically focal.

## 4. Clinical Examination Differentiators

A focused neurologic examination can substantially narrow the differential before electrodiagnostic testing. Strength testing should extend beyond ankle dorsiflexion to include toe extension, ankle eversion, ankle inversion, plantar flexion, and hip abduction.

Ankle inversion is particularly useful because tibialis posterior is predominantly L5 innervated but supplied by the tibial nerve. Preserved inversion therefore supports, but does not establish, an isolated common fibular lesion, whereas inversion weakness raises concern for a more proximal localization, particularly L5 radiculopathy or plexopathy. Inversion may remain preserved in fibular-division predominant sciatic neuropathy if tibial division fibers are relatively spared [3,5,6,10].

Hip abduction provides another useful examination target because gluteus medius receives superior gluteal rather than common fibular innervation and has substantial L5 contribution. Weakness may therefore provide additional support for an L5 level process, although it is not independently diagnostic. Plantar flexion and the Achilles reflex are generally spared in an isolated common fibular neuropathy but may be affected when a sciatic lesion substantially involves the tibial division or when S1 fibers are involved.

Sensory examination should be interpreted as supportive rather than definitive because peripheral nerve and dermatomal territories overlap. Common fibular neuropathy generally spares the plantar surface, while more extensive sciatic lesions may involve both fibular and tibial sensory territories. Radicular sensory symptoms may approximate a dermatomal distribution but are variable. A Tinel sign at the fibular head can support local nerve irritation but is insufficient by itself to establish localization [3,5,7].

## 5. Electrodiagnostic Approach

Electrodiagnostic testing provides physiologic evidence that can distinguish among competing localizations and should be designed to test the differential rather than simply confirm a presumed common fibular lesion. Nerve conduction studies and needle electromyography provide complementary information regarding lesion location, pathophysiology, severity, and chronicity.

Motor nerve conduction testing should evaluate the common fibular nerve, typically recording from extensor digitorum brevis and, when appropriate, tibialis anterior, with stimulation across the fibular head or neck to evaluate for focal slowing or conduction block. Recordings from extensor digitorum brevis and tibialis anterior can provide complementary information, particularly when one response is low or technically limited. Anatomic variants, including accessory deep fibular innervation, should be considered when an unexpected conduction pattern is encountered. Tibial motor conduction should also be considered when sciatic neuropathy, plexopathy, or more generalized peripheral nerve disease is in the differential.

Superficial fibular and sural sensory nerve action potentials help distinguish peripheral lesions from root disease. Sensory responses are typically preserved in radiculopathy because the lesion lies proximal to the dorsal root ganglion, whereas reduced sensory nerve action potentials support pathology distal to the dorsal root ganglion. Normal sensory responses do not, however, exclude a focal demyelinating common fibular lesion [5,6].

Needle electromyography should be tailored to the differential and should sample muscles representing different anatomic levels. Evaluation may include fibular-innervated muscles distal to the knee, such as tibialis anterior, fibularis longus, and extensor digitorum brevis; a tibial-innervated distal muscle; the short head of the biceps femoris; and L5-predominant muscles supplied by nerves outside the common fibular distribution, such as tibialis posterior or gluteus medius. Lumbar paraspinal muscles can provide additional evidence supporting radiculopathy when clinically appropriate.

Denervation restricted to common fibular-innervated muscles distal to the knee with sparing of the short head of the biceps femoris and appropriate nonfibular L5 muscles strongly supports, in the appropriate clinical context, a common fibular lesion at or distal to the fibular head. Abnormality of the short head of the biceps femoris supports localization proximal to its motor branch. Conversely, abnormalities involving paraspinal muscles and multiple L5 innervated muscles supplied by different peripheral nerves favor a root-level process [5,6,8,13].

Preferential fibular division involvement is well recognized in sciatic neuropathy. In a series of 100 patients, Yuen and colleagues found the peroneal division to be more severely affected than the tibial division in 64% of cases; tibialis anterior EMG was abnormal in 92% of patients, and the extensor digitorum brevis CMAP was low in amplitude or absent in 80% [13]. This preferential involvement helps explain why a proximal sciatic lesion may resemble a distal common fibular neuropathy when examination or needle sampling is restricted to muscles below the knee.

## 6. Imaging Correlation

Electrodiagnostic testing provides physiologic localization, whereas imaging can identify nerve morphology and structural causes of injury. High-resolution ultrasound and magnetic resonance imaging should therefore be viewed as complementary rather than competing modalities.

Ultrasound is particularly useful for evaluating the superficial common fibular nerve around the fibular head and neck. It permits assessment of nerve caliber and echotexture as well as dynamic evaluation and characterization of adjacent structural abnormalities. In a prospective cohort of 40 patients with clinically suspected common fibular neuropathy, Bignotti et al. reported a sensitivity of 90% and specificity of 92% for ultrasound, compared with 87.5% sensitivity and 85% specificity for conventional MRI, with diagnostic performance that favored ultrasound modestly [15]. These estimates were derived from a relatively small, selected cohort and should not be generalized to all forms of fibular neuropathy or to dedicated magnetic resonance neurography.

MRI provides a larger field of view and can evaluate deeper or more proximal pathology, surrounding soft tissues, and secondary muscle denervation changes. Magnetic resonance neurography may be particularly helpful when a proximal sciatic or plexus lesion is suspected or when the clinical and electrodiagnostic localization remains uncertain.

In traumatic common fibular nerve injury, ultrasound may also help determine nerve continuity and lesion level. A recent surgical cohort found ultrasound useful for identifying lesion location and characterizing nerve continuity relative to operative findings [16]. Because available comparative imaging studies are small and clinically heterogeneous, selection between ultrasound and MRI should be driven by the suspected lesion and the specific structural question rather than by a universal hierarchy of diagnostic accuracy.

## 7. Practical Localization Algorithm

A practical localization sequence begins by confirming true weakness of ankle dorsiflexion and assessing toe extension, ankle eversion, inversion, plantar flexion, and hip abduction. Upper motor neuron findings or a broader pattern of weakness should prompt consideration of central or generalized neurologic disease rather than immediate assumption of a focal peripheral lesion.

Sensory findings can then be used to refine, but not independently establish, localization. Electrodiagnostic testing should evaluate common fibular motor conduction across the fibular head, appropriate sensory responses, and tibial motor responses when indicated. Needle EMG should deliberately sample muscles capable of distinguishing an isolated common fibular lesion from sciatic, plexus, and L5 root pathology. Specifically, inclusion of the short head of the biceps femoris and appropriate nonfibular L5 muscles reduces the risk of misclassifying a proximal lesion as common fibular neuropathy.

Once physiologic localization has been established, imaging can be directed toward the suspected anatomic level. High-resolution ultrasound is well suited to focal superficial common fibular pathology, whereas MRI or MR neurography may be preferable when deeper, more proximal, or extensive disease is suspected.

## 8. Prognosis and Management

Management and prognosis are determined primarily by localization, etiology, severity, duration, and the degree of axonal injury. For compressive common fibular neuropathy, initial management commonly includes removal of external compression, modification of contributing activities or positioning, physical therapy, and an ankle-foot orthosis when necessary to improve gait safety [12,17].

Operative treatment may be appropriate for selected patients with persistent entrapment, nerve laceration, a surgically remediable compressive mass, or other structural pathology. The decision to pursue surgery should integrate the mechanism and duration of injury, examination, electrodiagnostic findings, imaging, and evidence of clinical recovery rather than rely on an isolated electrodiagnostic abnormality. Root, plexus, and sciatic lesions require treatment directed toward their underlying etiologies.

## 9. Conclusion

Foot drop is best approached as a neuroanatomic localization problem rather than a single diagnosis. Although weakness of dorsiflexion may initially appear to indicate common fibular neuropathy, lesions of the L5 root, lumbosacral plexus, and fibular division of the sciatic nerve can produce overlapping presentations. Careful examination of ankle inversion, eversion, plantar flexion, and hip abduction helps establish competing hypotheses before testing.

Electrodiagnostic evaluation can then distinguish these localizations by combining appropriately selected nerve conduction studies with needle examination of muscles above and below the knee and across different peripheral nerve distributions. Deliberate assessment of the short head of the biceps femoris is particularly helpful when distinguishing fibular division sciatic neuropathy from a lesion at the fibular head. Ultrasound and MRI provide complementary structural information after the likely level of injury has been identified. Together, targeted examination, electrodiagnostic testing, and appropriately selected imaging provide a systematic framework for localizing foot drop and directing subsequent etiologic evaluation and treatment.

## Figures and Tables

**Figure 1:**
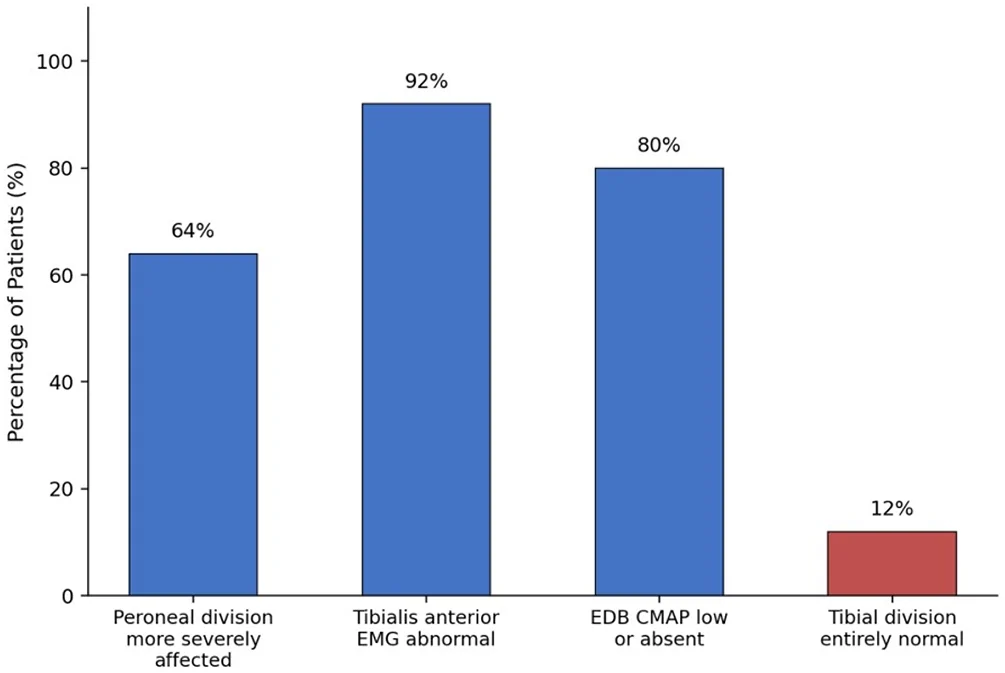
Electrodiagnostic findings in sciatic neuropathy (N=100 patients). Data are compiled from the published findings of Yuen et al. [13]. The disproportionate involvement of the peroneal division relative to the tibial division is the electrophysiological pattern that allows a proximal sciatic lesion to mimic an isolated fibular head neuropathy.

**Figure 2:**
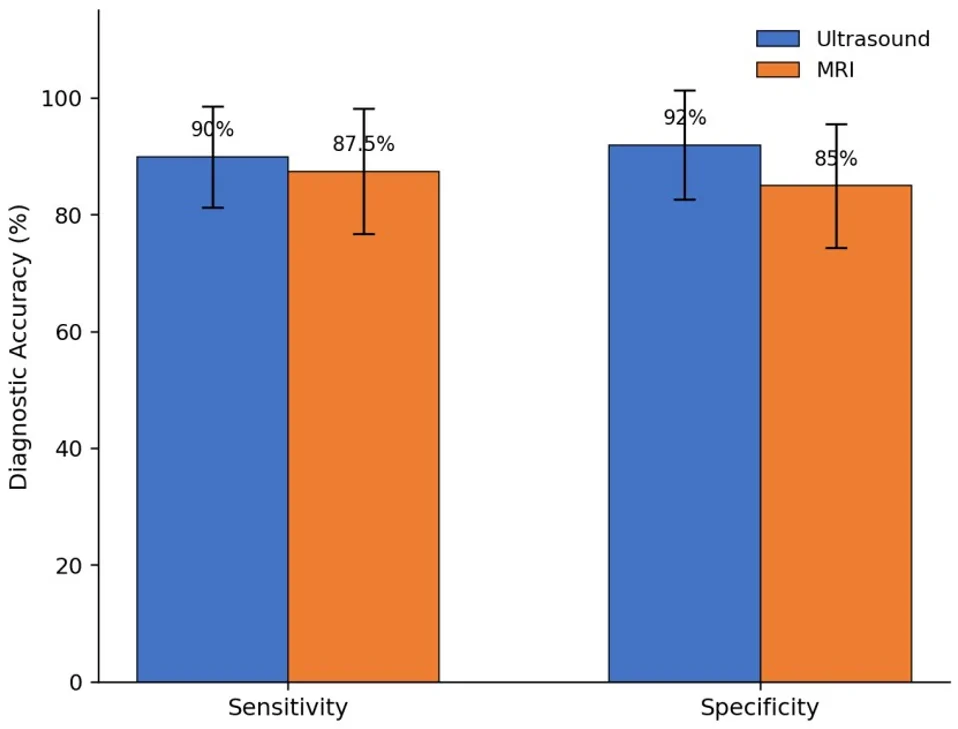
Ultrasound vs. MRI for Common Fibular Neuropathy at the Fibular Head. The bars show the comparison of diagnostic accuracy (sensitivity and specificity, with 95% confidence intervals) between ultrasound and MRI for common fibular neuropathy at the fibular head (N=40 patients). Data are compiled from the published findings of Bignotti et al. [15].

## Outstanding Questions

- How frequently is fibular-division-predominant sciatic neuropathy misclassified as isolated common fibular neuropathy in routine electrodiagnostic practice, and does misclassification affect treatment or outcomes?
- Can a standardized bedside examination combining ankle inversion, proximal L5 muscle testing, and sensory mapping reliably identify patients requiring expedited electrodiagnostic evaluation?
- What is the comparative diagnostic yield of ultrasound, conventional MRI, and MR neurography across specific etiologies and anatomic levels of neurogenic foot drop?
- Does routine sampling of the short head of the biceps femoris during electrodiagnostic evaluation of otherwise unexplained foot drop meaningfully improve diagnostic yield or alter management?

## Key Points

- **Foot drop is a localization problem.** Important neurogenic causes include L5 radiculopathy, lumbosacral plexopathy, sciatic neuropathy, and common fibular neuropathy.
- **Ankle inversion is a high yield bedside discriminator.** Preserved tibialis posterior strength supports an isolated common fibular lesion, whereas inversion weakness raises concern for a more proximal process, particularly L5 radiculopathy or plexopathy.
- **The short head of the biceps femoris is a useful proximal EMG muscle.** Abnormality supports a lesion proximal to its motor branch and can help distinguish fibular division sciatic neuropathy from common fibular neuropathy at the fibular head.
- **Sciatic neuropathy can mimic common fibular neuropathy.** Preferential involvement of the fibular division makes proximal muscle sampling important when distal abnormalities appear exclusively fibular.
- **Ultrasound and MRI are complementary.** Ultrasound is particularly useful for focal superficial lesions around the fibular head, whereas MRI provides a broader field of view for deeper or more proximal pathology.
- **Electrodiagnostic testing should evaluate competing localizations.** A complete study should not simply demonstrate common fibular dysfunction but should include muscles and sensory responses capable of distinguishing root, plexus, and sciatic lesions.
